# DNA Tumor Virus Regulation of Host DNA Methylation and Its Implications for Immune Evasion and Oncogenesis

**DOI:** 10.3390/v10020082

**Published:** 2018-02-13

**Authors:** Sharon K. Kuss-Duerkop, Joseph A. Westrich, Dohun Pyeon

**Affiliations:** 1 Department of Immunology and Microbiology, University of Colorado School of Medicine, Aurora, CO 80045, USA; joseph.westrich@ucdenver.edu; 2 Department of Medicine, University of Colorado School of Medicine, Aurora, CO 80045, USA

**Keywords:** DNA methylation, DNMT, antiviral immunity, immune evasion, herpesvirus, papillomavirus, KSHV, EBV, HBV, HPV

## Abstract

Viruses have evolved various mechanisms to evade host immunity and ensure efficient viral replication and persistence. Several DNA tumor viruses modulate host DNA methyltransferases for epigenetic dysregulation of immune-related gene expression in host cells. The host immune responses suppressed by virus-induced aberrant DNA methylation are also frequently involved in antitumor immune responses. Here, we describe viral mechanisms and virus–host interactions by which DNA tumor viruses regulate host DNA methylation to evade antiviral immunity, which may contribute to the generation of an immunosuppressive microenvironment during cancer development. Recent trials of immunotherapies have shown promising results to treat multiple cancers; however, a significant number of non-responders necessitate identifying additional targets for cancer immunotherapies. Thus, understanding immune evasion mechanisms of cancer-causing viruses may provide great insights for reversing immune suppression to prevent and treat associated cancers.

## 1. Introduction

Recent studies have revealed that DNA methylation is associated with many different diseases including microbial infections and cancers (reviewed in [1,2]). DNA methylation is a potent epigenetic mechanism to regulate gene expression without altering DNA sequences. Methylation of cytosines in CpG motifs frequently occurs in promoter regions but is also found in enhancers, insulators, gene bodies, transposable elements, and repetitive DNA elements (reviewed in [3]). DNA methylation is most dynamic in CpG islands near transcription start sites. CpG islands, which are typically hypomethylated, are DNA regions with a greater abundance of CpG dinucleotides compared to the remainder of the genome. Generally, promoter methylation represses gene transcription, while gene body methylation induces gene transactivation (reviewed in [3,4]). 

DNA methylation is catalyzed by six DNA methyltransferases (DNMTs) that have been characterized to date: DNMT1, DNMT2, DNMT3A, DNMT3B, DNMT3C, and DNMT3L. Among them, DNMT3A and DNMT3B produce multiple isoforms by an alternative promoter and an alternative splicing, respectively, for further regulation of their enzymatic activity (reviewed in [1]). Each DNMT has distinct functions in its role in gene expression regulation. DNMT1 is responsible for maintaining heritable DNA methylation by copying methylation patterns from a parental cell to a daughter cell shortly after mitosis (reviewed in [1]). Whereas DNMT2 is a tRNA methyltransferase [5], DNMT3A and DNMT3B are *de novo* methyltransferases that generate new methylation marks on unmethylated CpG DNA sites (reviewed in [1]). DNMT3C has been recently discovered in mice as a DNA methyltransferase involved in fertility [6]. DNMT3L is a catalytically inactive DNMT3 variant that interacts with and amplifies DNMT3A and DNMT3B activities [7,8,9,10]. While the mechanisms by which DNMTs methylate DNA have been well characterized, no specific DNA demethylase has been identified to date that reverses DNA methylation. Instead, it has been suggested that methylated cytosines are removed during DNA repair after the conversion of 5-methylcytosine to 5-hydroxymethylcytosine by the methylcytosine dioxygenases ten-eleven translocases (TET) (reviewed in [1,3]). 

Gene expression regulation by DNA methylation is intimately linked to chromatin arrangement (reviewed in [3,11,12]). In fact, chromatin structure is altered when DNA is methylated [11,13,14], and histone deacetylases (HDAC) interact with DNMT1 [15,16,17], DNMT3A [18,19], and DNMT3B [18]. Hypermethylated DNA is often associated with hypoacetylated histones and condensed chromatin for transcriptional repression [11,12]. 

Previous studies have suggested that DNA methylation functions as an antiviral defense mechanism by inactivating viral gene transcription and replication. It is well known that most endogenous retroviruses and retrotransposons in the human genome are inactivated by DNA hypermethylation [20,21]. Roulois et al. and Chiappinelli et al. have concurrently reported that treating colon and ovarian cancer cells with demethylating agents activates viral RNA transcription from dormant endogenous retroviruses and stimulates antiviral interferon (IFN) signaling, which subsequently activates antitumor immune responses [22,23]. DNA demethylation also activates retrotransposons and triggers antiviral signaling in zebrafish embryos [24]. In addition to endogenous retroviruses, the genomes of DNA viruses, such as human papillomavirus (HPV), herpes simplex virus 1 (HSV-1), adenovirus, and hepatitis B virus (HBV), are also frequently methylated and silenced in infected cells [25,26,27,28,29,30,31,32,33]. Interestingly, methylation of HPV DNA is commonly detected in infected cervical lesions, and its methylation levels correlate to disease progression in high-grade premalignant cervical lesions and cancer [34,35,36,37,38]. Similarly, methylation of HBV covalently closed circular DNA (cccDNA) markedly reduces viral gene transcription and genome replication during chronic infection [33,39]. 

Many viruses, particularly small DNA viruses, harbor genomic signatures indicating that they have evolved for millions of years to evade the antiviral effects of DNA methylation [40,41,42,43,44]. Our study has shown that the prevalence of CpG dinucleotides, the target motif of DNA methylation, is significantly lower in the genomes of papillomaviruses compared to other dinucleotide motifs [45]. These results suggest that gene expression regulation by DNA methylation may play a critical role in arms races between viruses and their hosts. 

To evade detection and restriction by the host immune response, viruses also employ various mechanisms to control gene expression related to immunity, including hijacking epigenetic machinery (reviewed in [46,47]). A recently described mechanism for viruses to epigenetically subvert host immunity is repression of immune-related gene expression by induction of DNA hypermethylation. In particular, DNA tumor viruses utilize this mechanism to manipulate host DNA methylation to alter expression of immune-related genes [48,49,50,51,52,53,54]. Indeed, several DNA tumor viruses have been found to regulate multiple DNMTs, suggesting that aberrant DNA methylation caused by viruses may be linked to virus-associated carcinogenesis [55]. Particularly in tumor virus infections, dysregulation of antiviral immune gene expression can have dual consequences. While a virus evades antiviral immune surveillance to establish a persistent infection, immune impairment induced by the virus can result in cancer cell evasion from antitumor immune responses during oncogenesis, as antiviral and antitumor immunity share similar immune mechanisms (reviewed in [56]). Since recent immunotherapies have shown promising efficacy to treat late-stage cancers [57,58,59,60,61], research regarding immune dysregulation by tumor virus-induced DNA methylation is of critical importance but largely understudied. Here, we discuss several compelling studies showing that DNA tumor virus regulation of host immune-related genes by DNA methylation contributes to cancer progression and is likely a result of virus-driven immune suppression to evade host antiviral responses.

## 2. DNA Tumor Viruses and DNA Methylation of Host Genes

**Figure 1:**
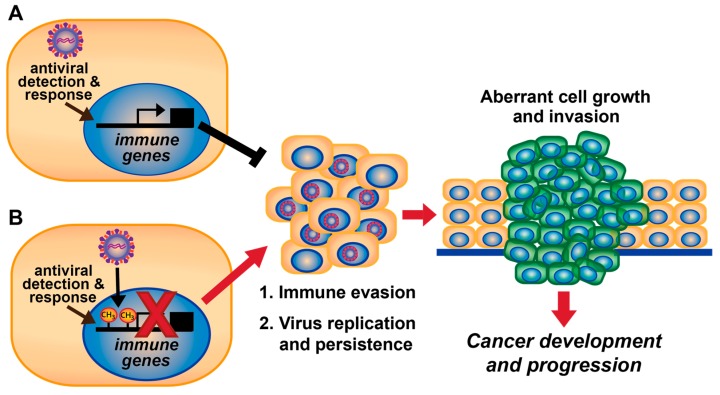
Model for DNA tumor virus-mediated DNA methylation to evade antiviral and antitumor immunity during viral persistence and carcinogenesis. (**A**) Cellular detection of viruses activates immune gene expression to induce an antiviral immune response. Proliferation of infected and neighboring cells can be blocked (black T bar) by immune-mediated apoptosis and/or cell cycle inhibition, which prevent cancer development. (**B**) DNA tumor viruses induce hypermethylation of immune genes that inhibit expression of antiviral immune genes (denoted by red “×”), resulting in immune evasion, which promotes (long red arrow) viral replication and persistence. Over long periods of time (multiple years), immune evasion and viral persistence can promote (short red arrow) cell proliferation and carcinogenesis. In addition, downregulation of immune gene expression by viral-induced DNA methylation may also contribute to host cell evasion of antitumor immune responses.

Hijacking DNA methylation machinery by DNA tumor viruses is likely a viral mechanism to promote virus replication by evading antiviral immunity. Immune suppression caused by aberrant DNA methylation over time may contribute to cancer development and progression associated with DNA tumor viruses (Figure 1). In fact, tumorigenesis is enhanced when antiviral immune responses are dampened [62,63,64,65] (reviewed in [56]). Kaposi’s sarcoma-associated herpesvirus (KSHV; also known as human herpesvirus 8), Epstein-Barr virus (EBV; also known as human herpesvirus 4), HBV, and HPV induce promoter methylation which downregulates expression of host immune-related genes, as will be discussed herein. Although here we focus on DNA tumor viruses, regulation of host immune genes by DNA methylation has also been demonstrated for the RNA virus human immunodeficiency virus (HIV) [66,67,68,69].

Many genome-wide methylome and transcriptome analyses have linked DNA tumor virus infection to the dysregulation of host gene hypermethylation during viral persistence and carcinogenesis. These DNA tumor viruses include gammaherpesviruses (EBV [70,71,72,73,74,75,76,77] and KSHV [78]), a hepadnavirus (HBV) [79,80], alphapapillomaviruses (HPV) [53,54,81,82,83,84,85], and polyomaviruses (simian virus 40 (SV40) [86,87,88,89], Merkel cell carcinoma virus (MCPyV) [90,91], JC virus (JCV) [92,93]) (Table 1). Frequently, virus-associated cancers show highly increased levels of DNMT expression [75,76,77,85,94,95,96,97,98,99]. In HBV-associated hepatocellular carcinoma (HCC), DNMT expression is inversely correlated with levels of tumor suppressor microRNAs (miRNAs), including miR-152 targeting DNMT1 [97] and miR-101 targeting DNMT3A [99]. Virus-induced DNA hypermethylation is commonly found on several tumor suppressor genes including *RASSF1A, p16* (also known as cyclin dependent kinase inhibitor 2A (*CDKN2A*)), *TP73, p21* (also known as *CDKN1A*), and retinoblastoma-associated protein (*pRb*) (Table 1). These findings suggest that induction of DNA methylation is likely a viral mechanism to promote cell proliferation that supports efficient viral replication, particularly for DNA viruses. Thus, downregulation of these tumor suppressors by promoter hypermethylation during virus infection could be a determinant of virus-driven tumorigenesis. We summarize host genes with diverse functions regulated by DNA methylation in cancers associated with DNA tumor viruses in Table 1. Additionally, DNA methylation-associated pathogenesis for EBV-associated gastric carcinoma (EBVaGC) [70] and HBV-associated HCC [79,80] have been previously reviewed in detail. Although definitive identification of particular hypermethylated genes that directly promote oncogenesis remains elusive, these studies have shown that increased DNA hypermethylation strongly correlates with disease progression of various virus-induced cancers. 

In contrast to virus-induced DNA hypermethylation, viruses can also decrease host DNA methylation to regulate host gene expression. DNA hypomethylation usually results in increased gene expression. For example, Kaposi’s sarcoma (KS) cell lines display hypomethylation of AXL receptor tyrosine kinase (*AXL*), which is linked to oncogenesis [100]. The KSHV viral FLICE-inhibitory protein (vFLIP) induces *AXL* expression potentially through *AXL* gene hypomethylation [100]. Alterations in DNA methylation status of particular genes by viruses may have profound effects on cancer development and progression.

## 3. Herpesviridae

### 3.1. Oncogenesis by Herpesviruses

Herpesviruses are large double-stranded DNA viruses that persistently infect their hosts, often for an entire lifetime. Herpesvirus infections generally do not cause any significant disease unless host immune responses are suppressed [101]. Herpesviruses have both lytic and latent cycles of infection. Once a lytic infection ensues, herpesviruses undergo a dormant cycle (latency) and occasionally reactivate from latency to undergo lytic replication. One genus of herpesviruses, gammaherpesviruses, such as KSHV and EBV, have oncogenic potential [101]. KSHV causes KS, primary effusion lymphomas (PEL) and multicentric Castleman disease in immunocompromised individuals (reviewed in [102]). EBV infection is associated with various lymphomas, including Burkitt’s and Hodgkin’s lymphomas, in addition to carcinomas of the nasopharynx and stomach (reviewed in [103]).

KSHV encodes multiple oncoproteins and oncogenic miRNAs that dysregulate host functions and promote cancer progression of endothelial and B cells (reviewed in [102,104]). A primary mechanism underlying KSHV-induced cancers is activation of angiogenesis by KSHV miRNAs and the oncoproteins vIRF3, K1, K8.1, K15, glycoprotein B (gB), latency-associated nuclear antigen (LANA), viral G-protein coupled receptor (vGPCR), vFLIP, and viral chemokines (reviewed in [104]). In addition, KSHV K1, vFLIP, LANA, and viral interleukin-6 (vIL-6) inhibit apoptosis in KSHV-infected cells to support cell proliferation as well as viral replication and persistence (reviewed in [102,104]). KSHV also activates the host cell cycle, cell proliferation, migration, and invasion, which contribute to viral oncogenesis (reviewed in [104]). LANA is consistently expressed in KSHV-positive cancers and known to induce angiogenesis and activate the host cell cycle by degrading p53 and stabilizing c-MYC (reviewed in [105]). Thus, KSHV has evolved mechanisms to modulate various aspects of host biology to drive oncogenesis.

EBV also encodes several proteins and miRNAs that promote transformation of B cells and epithelial cells, such as latent membrane proteins (*LMP1* and *LMP2*), EBV nuclear antigens (EBNA1-3, leader protein (*LP*)), BamHI fragment H rightward open reading frame 1 (*BHRF1*), BamHI A reading frame 1 (*BARF1*), and BamHI A rightward transcript miRNAs (*miR-BART*) (reviewed in [103,106]). Several miR-BARTs, BHRF1, and BARF1 block pro-apoptotic proteins in host cells (reviewed in [103,106]). EBV LMP activates oncogenic signaling including mitogen-activated protein kinase, c-Jun N-terminal kinase, phosphatidylinositol 3-kinase, and NF-κB pathways (reviewed in [106]). EBNAs stimulate cell proliferation through multiple mechanisms. EBNAs inhibit p53 and pRb tumor suppressors, hijack RBP-Jκ and NF-κB transcription factors to express oncogenes, usurp HDACs (reviewed in [107]), and induce DNA damage by activating recombinase-activating genes (*RAG1* and *RAG2*) [108]. 

### 3.2. Herpesviruses Modulate DNMTs

**Figure 2:**
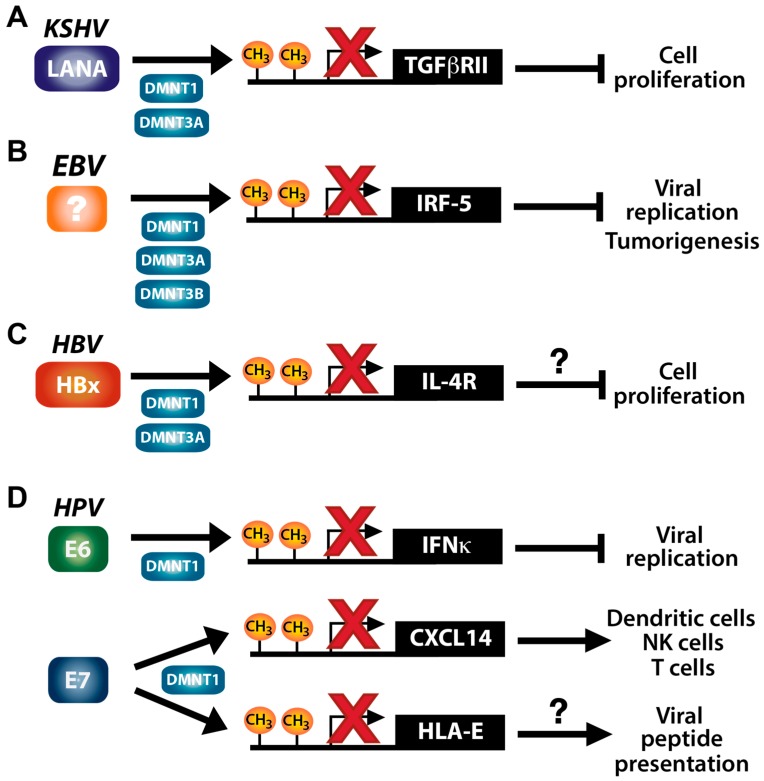
DNA tumor viruses that promote DNA hypermethylation of immune-related genes. (**A**) KSHV LANA; (**B**) an unidentified EBV protein; (**C**) HBV HBx; and (**D**) HPV E6 and E7 proteins upregulate the *DNMTs* shown to induce DNA methylation and transcription inhibition (indicated by red “×”) of the denoted immune-related genes. The outcome of immune gene suppression by the viruses promotes virus replication and host cell proliferation. Circled question marks indicate potential mechanisms that have not yet been fully defined.

Both KSHV and EBV have been shown to stimulate DNA hypermethylation of host genes, which likely contributes to virus-driven tumorigenesis. KSHV LANA interacts with DNMTs and recruits DNMT3A to host chromatin [109] (Figure 2A). DNMT3A mRNA expression is also increased in KSHV-infected cells, which likely results in DNMT3A-mediated repression of host genes [110]. KSHV vIL-6 enhances *DNMT1* expression levels and activity, resulting in global DNA hypermethylation in endothelial cells [111]. Treatment of vIL-6-expressing cells with the demethylating agent 5-aza-2′-deoxycytidine reduced aberrant cell proliferation and migration induced by vIL-6. These results suggest that vIL-6-induced host DNA hypermethylation increases host cell proliferation and migration [111]. 

EBV can infect and modulate DNMT levels in both epithelial and B cells. Expression of distinct DNMTs is regulated differently by EBV based on cell/tissue types and latency programs. EBV has three latency stages (I-III) determined by differential viral gene expression, which influences host gene expression, including DNMTs [112]. *DNMT1* and *DNMT3B* are upregulated in EBVaGC (latency I) [94,95] by LMP2A [94]; *DNMT1*, *DNMT3A*, and *DNMT3B* are upregulated in EBV-associated nasopharyngeal carcinoma (NPC; latency II) by LMP1 [76,77,113]; lastly, DNMT3A levels are increased in Hodgkin’s lymphoma cell lines (latency II) as well as lymphoblastoid cell lines derived from EBV-positive germinal center B cells (latency III) by an unknown viral gene [75] (Figure 2B). *DNMT1* and *DNMT3B* expression was shown to be downregulated in EBV-infected lymphoblastoid cells and Hodgkin’s lymphoma cell lines, which is distinct from that observed for EBVaGC and EBV-positive NPC [75]. Ectopic expression of EBV LMP1 reduces DNMT1 levels in lymphoblastoid cells [75]. LMP1 effects on DNMT1 levels in lymphoblastoid cells compared to EBVaGC and NPC could be due to differing EBV latency stages, which are dictated by distinct EBV gene expression patterns. Upregulation of *DNMT1* expression by EBV LMP2a in EBVaGC [94] may be compensatory for the lack of *LMP1* expression in latency I EBVaGC cells. Taken together, these results suggest that EBV upregulates different DNMT proteins in varying host cell/tissue types to induce hypermethylation of host genes (Figure 2B). In summary, DNA methyltransferase activity is altered in both KSHV- and EBV-infected cells, changing the landscape of promoter methylation and host gene expression.

### 3.3. KSHV and TGF-β Signaling

Downregulation of the transforming growth factor β (TGF-β) type II receptor (*TβRII*) by LANA-mediated promoter methylation contributes to development of KSHV-induced PEL [48] (Figure 2A). Upon binding of the ligand TGF-β, TGF-β type I receptor (TβRI) is recruited to and heterodimerizes with TβRII to initiate downstream signaling. TβR signaling contributes to embryo and organ development by regulating cell proliferation, differentiation, apoptosis, homeostasis, and other cellular processes (reviewed in [114,115,116]). Furthermore, the TβR signaling pathway plays important roles in cancer development and progression [117,118]. For example, TGF-β is considered a tumor suppressor as it inhibits proliferation of colon cancer cells [119], pancreatic ductal adenocarcinoma cells [120], and hepatocytes [118,121,122]. Interestingly, previous studies using cancer cell lines and patient tissue samples showed that loss of TGF-β signaling is often strongly correlated with hypermethylation of the *TβRII* promoter and poor prognosis for patients with different types of cancers [123,124,125,126,127,128,129]. TGF-β signaling is downregulated by KSHV, which stimulates cell proliferation and could promote cancer development [104]. KSHV-positive PEL cells were found to be unresponsive to TGF-β stimulation [48]. *TβRII* expression and TGF-β signaling activation was restored in KSHV-positive PEL cells by treatment with 5-aza-2′-deoxycytidine in combination with an HDAC inhibitor MS-275. Furthermore, reversing the epigenetic silencing of TGF-β signaling decreases cell proliferation and increases apoptosis [48]. These results suggest that downregulation of *TβRII* expression through KSHV-induced DNA methylation abrogates TGF-β signaling and drives transformation of KSHV-infected cells. 

Several studies have revealed that TGF-β signaling is detrimental to KSHV infection, as KSHV employs various mechanisms to avoid host restriction mediated by TGF-β signaling. These mechanisms include: (1) vFLIP and viral cyclin (vCyclin) activation of oncogenic host miRNAs that target SMAD family member 2 (SMAD2), a downstream component of TGF-β signaling [130]; (2) virally-encoded miRNA targeting of *TβRII* [131], *SMAD5* [132], and *thrombospondin 1*, a mediator of latent TGF-β activation [133]; (3) viral IFN regulatory factor 1 (vIRF1) binding to and inhibition of SMAD3-SMAD4 functions in the TGF-β signaling complex [134]; (4) viral K-bZIP disruption of the SMAD3 interaction with CREB-binding protein (CBP) [135], which is important for transcriptional activation of *TGF-β* [136,137,138,139,140]; and (5) cytokine receptor gp130 activation by vIL-6 that leads to downregulation of *TGF-β2* expression [141,142]. These findings strongly indicate that TGF-β signaling activation potently restricts productive KSHV infection. Taken together, epigenetic downregulation of *TβRII* by KSHV plays an important role for virus evasion of TGF-β signaling-mediated host restriction during virus persistence and disease progression (Figure 2A).

### 3.4. EBV and the Antiviral Protein IRF5

EBV induces promoter hypermethylation of IFN regulatory factor 5 (*IRF5*) during oncogenesis of Burkitt’s lymphoma and EBVaGC [49] (Figure 2B). IRF5 functions as both an antiviral signaling factor and tumor suppressor by inducing apoptosis in response to viral infection or DNA damage [143]. Activation of toll-like receptor (TLR)-myeloid differentiation primary response 88 (MyD88) signaling induces IRF5 phosphorylation, which translocates IRF5 into the nucleus and transactivates pro-inflammatory cytokines and DNA damage response genes [143,144,145]. IRF5 is a potent tumor suppressor. H-Ras transformed mouse embryonic fibroblasts (MEF) expressing *IRF5* do not develop tumors in vivo, whereas cells lacking IRF5 readily form tumors [143]. In this model, IRF5 tumor suppression is likely mediated through induction of apoptosis, as cells lacking IRF5 are resistant to apoptosis compared to *IRF5*-expressing cells [143]. Additionally, *IRF5* expression is decreased in breast cancer tissues, and overexpression of *IRF5* in breast cancer cell lines results in DNA damage-induced cell death and tumor suppression [146]. A previous study showed that EBV-induced promoter methylation and repression of *IRF5* transcription are linked to gastric carcinoma development [147]. Dong et al. demonstrated that hypermethylation of the *IRF5* promoter was 5-fold higher in EBVaGC cell lines compared to EBV-negative gastric carcinoma cell lines. In addition, *IRF5* expression in EBVaGC cells was rescued by treatment with a demethylating agent [49]. While the EBV protein that mediates *IRF5* DNA methylation remains unknown, these results imply that downregulation of *IRF5* expression by EBV blocks apoptosis of infected cells and contributes to cell transformation and oncogenesis. 

The antiviral activity of IRF5 was first demonstrated in vesicular stomatitis virus (VSV) and HSV-1 infections [143]. IRF5 inhibits proliferation of EBV-infected cells [148] and downregulates expression of both EBV *LMP1* mRNA [149] and the latency *BART* mRNAs [150]. In fact, LMP1 was recently shown to inhibit IRF5-mediated apoptosis during infection [151]. Interestingly, another study showed that although TLR7 signaling is activated during EBV infection, downstream *IRF5* expression is repressed by induction of an *IRF5* dominant-negative splice variant [152]. Overall, these results suggest that EBV has developed several mechanisms to block IRF5 induction of apoptosis in EBV infected cells. Thus, EBV-induced DNA methylation of *IRF5* may play an important role in evasion of host immunity during virus persistence and oncogenesis (Figure 2B). 

## 4. Hepadnaviridae

### 4.1. HBV Oncogenesis

HBV is a small, partially double-stranded DNA virus that infects hepatocytes and causes hepatitis, cirrhosis, and HCC. Nearly 4% of the worldwide population is chronically infected with HBV, which contributes to HCC being the fifth most common cancer worldwide (reviewed in [153]). HBV persistently infects hepatocytes as cccDNA, which is the genomic template for viral replication. HBV-driven HCC development is thought to occur through multiple oncogenic mechanisms including: (1) HBV DNA integration into the host genome; (2) cellular stress induced by accumulation of HBV surface antigen (HBsAg) in the endoplasmic reticulum; and (3) the multiple oncogenic functions of HBV X-protein (HBx). HBx interferes with proteasomal protein degradation, induces host miRNA expression, dysregulates host epigenetics, activates oncogenic signaling (e.g., Ras, Src and Wnt signaling), and stimulates the host cell cycle by inhibiting tumor suppressors such as p53 (reviewed in [153,154]). Altogether, persistent HBV infection presents a myriad of mechanisms that predispose cells to transformation. Here, we describe the roles of host DNA methylation in HBV infection and its impact on oncogenesis.

### 4.2. HBx Modulation of DNMTs

The HBV oncoprotein HBx upregulates expression of *DNMT1* and *DNMT3A*, which leads to promoter methylation and transcriptional repression of several tumor suppressor genes [96,155,156,157,158,159,160,161] (Figure 2C). HBx activates the host cell cycle by upregulating *DNMT1* through a positive feedback mechanism [158]. HBx represses expression of the cyclin dependent kinase (CDK) inhibitor *p16* by DNMT1-mediated promoter methylation. Downregulation of *p16* expression leads to cell cycle activation through inhibition of *pRb* and upregulation of *E2F1*. This ultimately results in increased DNMT1 levels and creates a positive feedback loop to further reduce *pRb* expression by *p16* promoter methylation [158]. HBx also promotes cell cycle progression through hypermethylation of other CDK inhibitors, *p21* and *p27* [157,162]. These results suggest that manipulation of the cell cycle by HBV HBx through enhanced DNA methylation may contribute to HCC development [157,158,161,162].

Several studies have demonstrated that expression of *DNMT1* and *DNMT3A/B* are upregulated in HBV-associated HCC tissues compared to adjacent normal liver tissues from patients [96,97,98,99]. On the other hand, studies using mice expressing HBx in hepatocytes revealed roles for HBx in DNA hypomethylation, which involve altered *DNMT* expression or promoter binding and may promote the development of HCC [163,164,165]. HBx repressed expression of *DNMT3A* and *DNMT3L* in HBx-expressing mice by binding their promoters in conjunction with HDAC1, which leads to global hypomethylation of CpG regions in the host genome [163]. Additionally, epithelial cell adhesion molecule (*EpCAM*) expression is upregulated in HBV-associated HCC by HBx-induced hypomethylation of the *EpCAM* gene by an uncharacterized mechanism, which involves DNMT3L [164]. Despite the well established role of DNMT3L in enhancing DNMT3A and DNMT3B activity [7,8,9,10], DNMT3L can also negatively regulate DNA methylation by competing with DNMT3A and DNMT3B binding to polycomb-repressive complex 2 (PRC2) to prevent de novo DNA methylation at histone 3 lysine 27 trimethylation (H3K27me3) sites [166]; this may explain the role of DNMT3L in HBx-mediated upregulation of *EpCAM* expression. In addition, the cyclooxygenase-2 (*COX-2*) promoter is hypomethylated in HBV-positive cells with reduced binding of DNMT3B to the *COX-2* promoter. HBx transgenic mice display elevated *COX-2* expression as compared to mice lacking HBx, indicating that HBx is critical for increasing *COX-2* expression in hepatocytes [165]. These results suggest that HBV HBx employs multiple mechanisms to induce or inhibit DNA methylation on different genes during hepatocarcinogenesis.

### 4.3. HBV and IL-4R Signaling

In addition to hypermethylation and downregulation of the tumor suppressors *p16*, *p21* and *p27*, HBx also induces methylation of the IL-4 receptor (*IL-4R*) gene, leading to downregulation of its expression [50]. The ligand of IL-4R, IL-4, is an anti-inflammatory cytokine that suppresses host cell growth and induces apoptosis [167]. IL-4R signaling primarily functions in hematopoietic cells; however, its activity has been observed in hepatocytes as well [168,169,170]. Interestingly, Zheng et al. found that expression of several genes downregulated by HBx is restored when DNA methylation is inhibited by treatment with 5-aza-2′-deoxycytidine [50]. The authors further revealed that HBx binds DNMT1 and DNMT3A. Notably, HBx binds the *IL-4R* promoter to facilitate its DNA methylation and silencing of *IL-4R* expression is DNMT3A-dependent [50]. These results indicate that *IL-4R* expression is repressed by HBx in HBV-infected hepatocytes through promoter methylation (Figure 2C). 

IL-4R signaling limits HBV infection [171,172]. Activation of IL-4R signaling in HBV-infected hepatocytes inhibits viral replication and reduces *HBsAg* and HBV e antigen (*HBeAg*) expression. IL-4 represses expression of *C/EBPα*, a transactivator of the HBV genome core promoter, to inhibit HBV replication [171]. Consistently, production of both HBsAg and HBeAg is decreased by IL-4 treatment [172]. Altogether, these results suggest that HBV replication and viral gene expression is inhibited by IL-4. Thus, downregulation of *IL-4R* expression by HBx-induced promoter methylation is likely an immune evasion mechanism of HBV [50]. Since IL-4-mediated signaling is pro-apoptotic [173], HBV downregulation of *IL-4R* expression via promoter methylation may contribute to cell proliferation and HCC development.

## 5. Papillomaviridae

### 5.1. HPV Oncogenesis

HPVs are small double-stranded DNA viruses that infect cutaneous and mucosal keratinocytes. While infection with low-risk HPV genotypes (e.g., HPV6 and -11) leads to development of benign skin lesions such as warts, several high-risk genotypes (e.g., HPV16 and -18) are causally associated with cervical, anogenital, or head and neck cancers (HNC). HPV associated cancers account for over 5% of all cancers worldwide [174]. While the majority of initial HPV infections are cleared within a few years, about 10% of infected people establish persistent HPV that likely exists for their lifetime [175,176]. Persistent infection with high-risk HPV genotypes and continuous expression of the HPV oncogenes, E6 and E7, are required for HPV-associated cancer progression and maintenance [177,178]. E6 and E7 contribute to cancer progression through various oncogenic mechanisms including inactivation of the tumor suppressors p53 and pRb, respectively [179]. Recent studies have suggested that dysregulation of DNA methyltransferase activity may also affect HPV-associated carcinogenesis.

### 5.2. HPV Modulation of DNMTs

HPV E6 and E7 enhance promoter methylation by upregulation of *DNMT1* expression through p53 degradation and a direct interaction with the DNMT1 protein, respectively [180,181] (Figure 2D). Our studies have shown that dysregulation of host DNA methylation by HPV16 E7 is associated with host immune suppression during HPV-associated cancer progression [53,54,182]. Interestingly, a recent clinical trial revealed that treatment with DNA methylation inhibitors suppressed HPV-positive HNC growth. Notably, HPV-positive HNC is more sensitive to treatment with the DNA demethylating agent 5-aza-2′-deoxycytidine compared to HPV-negative HNC [183]. These findings suggest that HPV dysregulation of DNA methylation can be reversed using demethylating agents as a targeted therapy for HPV-associated cancers. Here, we discuss several immune genes regulated by HPV through promoter hypermethylation.

### 5.3. High-Risk HPV and IFNκ Signaling

Dysregulation of immune-related gene expression by high-risk HPV-mediated DNA methylation was first demonstrated with IFN-kappa (*IFNκ*) [51,52] (Figure 2D). IFNκ is a type I IFN that is constitutively expressed in human keratinocytes, the natural host cell type for HPV infection [184]. IFNκ is an antiviral factor that restricts HPV replication in keratinocytes [185]. Previous studies showed that *IFNκ* expression is significantly downregulated in cells harboring high-risk HPV genomes (HPV16, -18 or -31) or expressing HPV16 E6 [51,52]. *IFNκ* expression in HPV-positive cells is restored by treatment with the demethylating agent 5-aza-2′-deoxycytidine, indicating that HPV induces methylation of *IFNκ* to reduce its expression. HPV16 E6, but not E7, is necessary and sufficient for induction of *IFNκ* promoter methylation [51,52]. Consistently, HPV16-positive cervical intraepithelial neoplasia and cervical cancer tissues are devoid of *IFNκ* expression, whereas HPV16-negative normal mucosal tissues display strong *IFNκ* expression [51]. Furthermore, ectopic expression of *IFNκ* in HPV16-positive cells restores antiviral signaling as determined by induction of IFN-stimulated gene expression and suppression of VSV replication. This indicates that the downstream signaling of IFNκ is still intact despite decreased *IFNκ* expression by HPV16 E6 [51]. These results suggest that high-risk HPV E6 interferes with expression of type I IFN to promote HPV persistence in host cells. 

### 5.4. High-Risk HPV and CXCL14 Expression

In previous gene expression studies using cervical and HNC patient tissue samples, we have revealed that numerous immune-related genes are dysregulated in HPV-positive cancers compared to normal tissue and HPV-negative cancers [54,186,187]. To determine if HPV directly affects expression of these immune-related genes, we recently performed a global gene expression analysis using normal keratinocytes with and without the HPV16 genome [53,54]. The two most downregulated groups of genes were those involved in immune regulation and extracellular matrix organization. Furthermore, many of these immune-related genes were specifically downregulated by the HPV16 oncoprotein E7, which was previously suggested to suppress antitumor immune responses [53,54,188]. Interestingly, our recent study showed that HPV16 E7 significantly downregulates the chemokine (C-X-C motif) ligand 14 (*CXCL14*) through promoter hypermethylation [54]. 

CXCL14 is a relatively novel chemokine, and its native receptor is still unidentified. CXCL14 inhibits angiogenesis and directly recruits several types of immune cells such as dendritic, natural killer (NK), and T cells [54,189,190]. We and other groups have shown antitumor activity of CXCL14 in cancers of the lung, head and neck, colon, and liver [54,191,192,193,194,195]. Consistently, the levels of *CXCL14* expression are reduced in these and other cancers [54,187,193,195,196,197]. Our study revealed that HPV16 E7 is responsible for *CXCL14* downregulation by facilitating hypermethylation of the *CXCL14* promoter, which is reversed by 5-aza-2′-deoxycytidine treatment [54] (Figure 2D). Restoration of *CXCL14* expression in HPV-positive cancer cells significantly increases NK and T cell recruitment and dramatically suppresses tumor cell growth in vivo. These results suggest that CXCL14 is a tumor suppressing chemokine, which is downregulated by HPV E7-induced promoter hypermethylation [54]. Similarly, *CXCL14* is also downregulated by promoter methylation in HCC cells and patient tumors [195,198]. Consistently, ectopic expression of *CXCL14* in HCC cells decreases colony formation, cell viability, cell invasion, and tumor growth in vitro and in vivo [195]. As HBV infection is a major driver of HCC, this suggests that HBV might similarly downregulate *CXCL14* expression during persistence and HCC development. 

Although a direct antiviral role of CXCL14 has not been demonstrated, CXCL14 may play a protective role at the cutaneous and mucosal skin layers to prevent HPV infection. CXCL14 is highly expressed in normal keratinocytes and structurally similar to antimicrobial proteins such as defensins [199], which restrict HPV infection [200,201]. Taken together, these results imply that downregulation of *CXCL14* by HPV E7-induced DNA methylation to evade host immunity contributes to suppression of host antitumor immune responses during HPV persistence and cancer progression.

### 5.5. High-Risk HPV and HLA-E Expression

We have recently discovered that high-risk HPV E7s, but not low-risk HPV E7s, downregulate HLA-E expression in keratinocytes by promoter hypermethylation, as shown by restoration of *HLA-E* expression using a demethylating agent (Figure 2D) [53]. HLA-E is a non-classical major histocompatibility complex I (MHC-I) protein that presents T cell epitopes on the cell surface and regulates NK and CD8^+^ T cell activation (reviewed in [202]). Peptide presentation, usually of self antigens, to NK cells by HLA-E inhibits NK cell-mediated cytolysis; however, pathogen antigen presentation by HLA-E typically prompts the killing activity of CD8^+^ T cells, including NK T cells, a subset of CD8^+^ T cells (reviewed in [202,203]). Therefore, downregulation of *HLA-E* by high-risk HPV E7 [53] implies that HLA-E may present HPV peptides to CD8^+^ T cells, resulting in elimination of infected cells. There is precedence for HLA-E presentation of viral peptides, but a clear antiviral mechanism has not been studied. A recent study showed that HLA-E interacts with and presents a conserved HIV-1 envelope peptide to activate NK cells to kill virus-infected T cells [204]. Thus, it is possible that downregulation of *HLA-E* expression by HPV-induced promoter hypermethylation is a viral mechanism of avoiding immune detection and cell-mediated cytotoxicity by decreasing viral peptide presentation to CD8^+^ T cells. This may lead to viral persistence and HPV-associated cancer development. 

## 6. Other DNA Tumor Viruses

In addition to the viruses described above, other DNA tumor viruses alter promoter methylation of host genes. Adenovirus-5 E1A upregulates *DNMT1* expression and also directly interacts with DNMT1 protein. Interestingly, a transformation-deficient E1A mutant that cannot bind DNMT1 abrogates virus-induced DNA methylation [181]. This suggests that the interaction of E1A with DNMT1 and induction of DNA methylation is linked to cellular transformation. Additionally, infection with the polyomavirus SV40 stabilizes DNMT activity and increases host DNA methylation in immortalized fibroblasts by unknown mechanisms [205]. Moreover, SV40 antigens are associated with aberrant DNA methylation in tumor tissues (Table 1) [86,87,88,89], suggesting that its stabilization of DNMT activity might promote tumor progression. Similarly, the presence of antigens from two other polyomaviruses, Merkel cell polyomavirus and JC virus, correlated with DNA hypermethylation in tumor tissues [90,91,92,93]. Polyomavirus-induced DNA hypermethylation is often found on tumor suppressor genes (Table 1). Altogether, these results suggest that viral-induced host DNA methylation may be a common mechanism to repress host gene expression to facilitate persistent viral infection and potentiate virus-induced cancer progression (Figure 1).

## 7. Conclusions

Recent studies in virus-driven dysregulation of host immune-related gene expression through DNA methylation presents a novel viral mechanism to inhibit immune responses. This field is largely understudied, and several important questions remain: (1) Is alteration of host DNA methylation a major mechanism generally employed by diverse viruses, including RNA viruses, to regulate immune responses? (2) Are specific antiviral immune genes prone to virus-driven DNA methylation? (3) Are there specific hot spots in host genomes that viruses target utilizing DNA methylation to alter gene expression? Parallel analyses of global gene expression and the cellular methylome altered by virus infection may be useful to determine whether viral evasion of host immune responses is associated with aberrant DNA methylation induced by diverse viruses. These analyses would also reveal whether certain immune-related genes are commonly targeted by different viruses to evade host immunity.

One factor that increases the probability of DNA methylation in a promoter region is the presence and abundance of CpG islands. Additionally, DNA methylation site specificity can be facilitated through multiple mechanisms: (1) specific transcription factors and DNA binding proteins that recruit DNMTs to distinct genomic regions; (2) DNMT interactions with HDACs to enhance chromatin packaging and gene silencing; (3) three-dimensional DNA structural changes that alter DNA binding protein accessibility; and (4) nucleosome stability and positioning in the nucleus (reviewed in [11,12]). However, the exact signals or mechanisms that drive specificity of genes affected by DNA methylation are mostly unknown. Defining how particular genes are targeted by virus-induced DNA methylation would significantly impact our understanding of cellular gene regulation not only by viruses, but also different cellular stimuli or processes, through DNA methylation. In addition, the majority of viruses discussed above upregulate *DNMT* expression and/or activity; however, these viruses can also modulate other factors involved in epigenetic reprogramming linked to DNA methylation, such as histone modifications (reviewed in [206]). This may provide additional means to control host gene transcription through DNA methylation independently of *DNMT* upregulation. It is of interest to understand how cellular gene transcription is affected by the interplay between DNA methylation and other epigenetic factors. For instance, KSHV-induced downregulation of *TβRII* was not fully reversed using a demethylating agent or HDAC inhibitor alone, but a combination of the two fully restored *TβRII* expression [48]. These results suggest that some host genes are not silenced simply through promoter hypermethylation or histone deacetylation alone, and therefore, viruses may have evolved mechanisms to ensure host gene downregulation through multiple epigenetic modifications. Similar transcriptional regulation occurs in cells to regulate gene expression, but the mechanisms remain elusive [3]. Using viruses, or viral proteins mediating host epigenetic changes, could also be useful in dissecting how DNA methylation alters chromatin structure, or vice versa. In addition, viral studies on transcriptional regulation by DNA methylation and chromatin modifications may help reveal which epigenetic change initiates gene silencing, as this is still largely unclear. 

Further mechanistic understanding is necessary to define the role and result of virus-driven aberrant DNA methylation of particular genes in oncogenesis. These studies would be of great interest and may provide useful targets for novel treatments for these virus-associated cancers. Since many DNA tumor viruses stimulate DNA methylation of host genes, including tumor suppressors and immune regulators, demethylating agents could be used to treat virus-associated cancers. In fact, 5-aza-2′-deoxycytidine treatment of HPV-positive HNC cells resulted in cell cycle arrest, p53-dependent apoptosis, activation of IFN signaling, and inhibition of metastasis [183]. 5-aza-2′-deoxycytidine treatment also decreased HPV gene expression within infected cells, which may result from the aforementioned effects of 5-aza-2′-deoxycytidine treatment on cell proliferation and IFN signaling [183]. Additionally, recent studies have shown that inhibition of DNA methylation significantly induces antitumor immune responses in colon and ovarian cancers [22,23]. Treatment with 5-aza-2′-deoxycytidine reactivates endogenous retroviruses that are recognized by cellular innate immune receptors and stimulate antiviral IFN responses [22,23]. Accordingly, demethylating drugs are generally being considered for cancer treatment in combination with other therapeutics to combat aberrant DNA methylation in oncogenesis (reviewed in [1]) and may be a highly effective strategy to treat virus-associated cancers. Therefore, a better understanding of virus-mediated dysregulation of host DNA methylation is of critical importance.

## Figures and Tables

**Table 1:** DNA methylation associated with DNA tumor virus-induced carcinomas.

Virus	Cancer Type	Tumor Tissue (TT) or Cell Line (CL)	Methylated DNA/Gene	Reference
*Herpesviridae*				
EBV	Gastric carcinoma			Reviewed in [70]
	NPC	TT, CL	*miR-31*	[71]
		TT	*DAPK*, *RASSF1A*, *p16* (*CDKN2A*)	[72]
		TT	*RASSF1A*	[73]
		CL	*Retinoic acid receptor-β2* (*RAR-β2*)	[76]
		CL	*E-cadherin*	[77]
	Burkitt’s lymphoma (BL)	CL	4712 differentially methylated genes	[74]
	Germinal center (GC) B cell malignancies, Hodgkin’s lymphoma (HL)	TT (GC) CL (HL)	1745 DMPs	[75]
KSHV	Primary effusion lymphoma	CL	*p16* (*CDKN2A*)	[78]
*Hepadnaviridae*				
HBV	HBV-associated HCC			Reviewed in [79,80]
*Papillomaviridae*				
HPV	Head and neck SCC	TT	*NSD1*, *NOTCH1*	[81]
	Cervical squamous intraepithelial lesions	TT	*SIM1*, *DLX4*	[82]
	E6/E7 immortalized keratinocytes	CL	*hTERT*, *miR124-2*, *PRDM14*, *FAM19A4, SFRP2*, *PHACTR3*, *MAL*, *CYGB*, *ROBO3*	[83]
	HPV16/18 keratinocytes, cervical cancer cells	CL	*hTERT*	[84]
	Head and neck SCC, cervical carcinoma	TT	*CXCL14*	[54]
	HPV16/18 immortalized keratinocytes	TT	*HLA-E*, *CCNA1*, *TERT*; 5190 DMPs	[53]
	SCC	CL	75 differentially methylated genes	[85]
*Polyomaviridae*				
SV40	Diffuse large B cell lymphoma	TT	*DAPK*, *CDH1*, *GSTP1*, *p16* (*CDKN2A*), *SHP1*	[86]
	Non-Hodgkin’s lymphoma/leukemia	TT	*CDH1*, *CDH13*, *CRBP*, *p16* (*CDKN2A*), *DAPK*, *DcR1*, *DcR2*	[87]
	Malignant mesothelioma	TT	*RASSF1A*	[88,89]
	Lung adenocarcinoma	TT	*RASSF1A*	[88]
MCPyV	Merkel cell carcinoma, small cell lung cancer	TT	*RASSF1A*	[90,91]
JC virus	Gastric carcinoma	TT	*p16* (*CDKN2A*), *p14*	[92]
	Colorectal cancer	TT	*hMLH1*, *PTEN*, *RUNX3*, *p16* (*CDKN2A*)	[93]

NPC: Nasopharyngeal carcinoma; SCC: Squamous cell carcinoma; DMP: Differentially methylated position.
